# Type II Autoimmune Hepatitis and Small Duct Sclerosing Cholangitis in a Seven Years Old Child: An Overlap Syndrome?

**DOI:** 10.5812/hepatmon.14452

**Published:** 2013-12-06

**Authors:** Andrea Domenico Pratico, Stefania Salafia, Patrizia Barone, Mario La Rosa, Salvatore Leonardi

**Affiliations:** 1 Department of Pediatrics, University of Catania, Catania, Italy; 2 Unit of Pediatrics, Lentini Hospital, Lentini, Italy

**Keywords:** Hepatitis, Autoimmune, Primary sclerosing cholangitis, Liver Diseases, Anti-Liver Kidney Microsome Antibody

## Abstract

**Introduction:**

Autoimmune hepatitis is an inflammatory disease with multifactorial ethiopatogenesis, characterized by lympho-monocytic infiltration of liver, presence of serum autoantibodies (ANA, SMA, LKM-1) and high levels of immunoglobulins. Overlap syndromes are defined as the association of autoimmune hepatitis with cholestatic diseases such as primary biliary cirrhosis and primary sclerosing cholangitis. The boundaries of these syndromes as distinct pathological entities are still matter of debate and they could be part of a major liver autoimmune disease. Furthermore, cholestatic diseases may present even with atypical features (AMA-negative primary cirrohosis, primary sclerosing cholangitis with normal cholangiography).

**Case Presentation:**

We herein describe a case of a 7 year-old child affected by an overlap syndrome between type 2 autoimmune hepatitis and small duct primary sclerosing cholangitis. Although characterized by a severe onset, the disease showed a good response to treatment with prednisone and azathioprine.

**Conclusions:**

The association of type 2 autoimmune hepatitis and small duct primary cholangitis has been rarely reported in literature and this report adds new data on this still unclear entity.

## 1. Introduction

Autoimmune hepatitis (AIH) is an inflammatory disease with a multi-factorial etio-pathogenesis characterized by peri-portal lymphomonocytic infiltration of liver, liver-specific and/or non-organ-specific autoantibodies, hyper-gamaglobulinemia (1). AIH may be associated with different cholestatic diseases such as primary biliary cirrhosis and primary sclerosing cholangitis, resembling findings of other immune-mediated liver diseases. These associated phenotypes have been designated 'overlap syndromes' although the validity of these syndromes as distinct pathological entities remains unclear (2).

We describe a case of type 2 AIH associated with a small duct autoimmune cholangitis in a 7-year girl to add new data on this rare association whose bonders are still uncertain in childhood.

## 2. Case Presentation

The patient is a seven year old Sri-Lankan girl. In January 2012 she contracted an upper airways infection with fever and after one week she presented yellow discoloration of eyes and acholic stools. Blood investigations showed an increase of total bilirubin (19.5 mg/dl, direct 9.4 mg/dl, and indirect 10.1 mg/dl), transaminases (AST 1216 U/L, ALT 1022 U/L) and alkaline phosphatase (524 U/L). An abdominal scan showed irregular liver surface with inhomogeneous structure, compatible with chronic liver disease. In March, the child was admitted at our unit: general conditions were good, weight was 12.7 kg (25th pc), height 92.2 cm (25th pc); she showed yellow eyes discoloration and hepatomegaly. Laboratory tests showed elevated ESR (51 mm/h), LDH (1567 U/L), alkaline phosphatase (713 U/L), transaminases (AST 1391 U/L, ALT 1405 U/L), γ-GT (294 U/L) and IgG (2110 mg/dl). Liver kidney antibodies (LKM) were strongly positive (1:640). Other test, including Anti-native DNA antibodies, ASMA, AMA, EMA and tTG, were normal. A liver biopsy showed enlargement of portal spaces correlated to a lymphocytic infiltrate with plasma-cells, neutrophiles and eosinophiles. This process exceeded the limiting membrane with piecemeal necrosis interesting the epithelium of the bile ducts. Periportal and portal fibrosis (onion-like), vacuolar degeneration of hepatocytes with formation of binucleate cells and pseudorosettes and signs of lobular inflammation with formation of apoptotic bodies were also present, together with a reduction of the number of biliary ducts. A magnetic resonance cholangiography was then performed, showing normal duct anatomy and no signs of large duct sclerosing cholangitis. For this reason a diagnosis of overlap syndrome of type 2 AIH and small duct cholangitis was performed.

Treatment with prednisone at a dose of 15 mg twice daily (2 mg/kg/day) was followed by a general improvement. After two months the patient presented a mild but persistent increase of transaminases and alcaline phosphatase. There were also signs of hypercortisolism and hypertension, and for this reason a gradual reduction of prednisone to 10 mg/day was performed and azathioprine at a dose of 1.5 mg/kg/day was introduced. Two weeks later the liver enzyme levels returned to the normal range.

## 3. Conclusions

Our patient presented a type 2 AIH and biochemical (high direct bilirubin, alkaline phosphatases and γ-GT serum levels) and histological features of cholestatic liver disease suggestive of small duct PSC.

AIH is an inflammatory disease characterized by hepatic cells damage associated with hypergammaglobulinemia and the presence of auto-antibodies. In North Europe the incidence is 1.9 cases per 100,000 per year (higher in female sex) and all ages and ethnic groups are interested (1). 

The diagnosis of AIH is based on exclusion of other causes of chronic liver disease such as genetic diseases like α1-antitrypsin deficiency, hemochromatosis, Wilson's disease, viral infections (HAV, HBV or HCV), and drug hepatotoxity. The diagnostic criteria include a specific scoring system defined by the International Autoimmune Hepatitis Group in 1999 (3) and simplified in 2008 by Hennes and coll (4).

Two forms of AIH are usually distinguished. Type I is more common in the second decade of life and between 45 and 70 years (5). It is associated with antinuclear antibodies (ANA) and/or anti-smooth muscle antibodies (ASMA). Type II is characterized by serum liver kidney microsomal anti-1 (LKM1) positivity. It is the less common form of AIH and it may present like a fulminant hepatitis, especially in younger patients or with a rapid evolution toward cirrhosis (6). Histological biliary changes, including bile duct damage, acute and/or chronic cholangitis, and biliary pattern of periportal hepatitis, have also been noted in 31% of children with AIH (7).

Overlap syndromes are characterized by the histological abnormalities of AIH and biliary tract involvement, which are mainly primary biliary cirrhosis (PBC) or primary sclerosing cholangitis (PSC). PBC is always characterized by bile duct injury, AMA positivity and increase of alkaline phosphatase and γ-GT, while PSC is typically characterized by AMA-negativity and overall by cholangiography abnormalities and it is more common in childhood: for this age it has been also proposed the definition “autoimmune sclerosing cholangitis”, to designate the overlap between AIH and PSC (8).

However these distinct pathological entities sometimes are uncertain. In fact patients with AIH and histological features of bile duct injury or loss, with AMA negativity, absence of large bile duct injury or inflammatory bowel disease, normal cholangiography have also been described (2). These patients represent a different syndrome designated as ‘‘autoimmune cholangitis”, or they may be variants of the two above reported overlap syndromes and designated as ‘‘AIH with AMA-negative PBC’’ or ‘‘AIH with small duct PSC’’ (2).

The histological findings of AIH include a dense mononuclear and plasma-cells infiltration of the portal areas, which expands into the liver lobule, destruction of the hepatocytes at the periphery of the lobule with erosion of the limiting plate (‘‘interface hepatitis’’).Connective tissue collapse results from hepatocyte death and expands from the portal area into the lobule (‘‘bridging collapse’’) with hepatic regeneration and ‘‘rosette’’ formation (9). In our patient we found loss or proliferation of interlobar ducts due to biliary tract injuries and peri-ductal inflammation and onion-skin type fibrosis, all findings diagnostic of a small duct PSC (10).

Our case is very interesting for different aspects such as the very early age of onset and the coexistence of type II AIH and PSC. Differently from our patient, in the experience of Olssen and coll (10), the median age of onset of AIH with large or small tract PSC was 27-28 years (ranging 15-69 years) and it was often associated with other autoimmune disorders (ulcerative colitis, Crohn’s disease, celiac disease, non-specific colitis, diabetes). Furthermore, LKM positivity is rarely reported in this overlap syndrome (2, 11). In particular, in the report of Gregorio et al. (8), only one patient of 27 with AIH-ASC presented LKM positivity, while 20/27 presented ANA and/or SMA.

The diagnosis of the overlap syndrome of AIH with PSC requires predominant features of AIH by clinical judgment or the scoring system of the IAIHG (3), absence of AMA, and features of cholestasis including jaundice, high serum alkaline phosphatase and γ-GT levels, or histological changes that suggest bile duct injury or loss (2, 11). The diagnosis is usually confirmed by abnormalities in cholangiography. On the contrary, as in our case, a normal cholangiogram associated with histological data of biliary tract involvement leads to a diagnosis of “small duct PSC” (10).

Therapy of overlap forms does not differ from AIH alone, although in some cases with persistent hyperbilirubinemia ursodeoxycholic acid may be helpful. In children the treatment is usually performed at the diagnosis. It includes prednisone at 2 mg/kg/day (up to 60 mg/day), alone or in combination with azathioprine; Maintenance regimen is reached by prednisone decrease to 0.1-0.2 mg/kg/day or 5 mg/day, alone or in combination with azathioprine. This regime is maintained for at least 1-2 years after the normalization of liver parameters, the absence of clinical signs, and no evidence of active inflammation at biopsy.

Most patients show an improvement of liver tests after 2-4 weeks of treatment with laboratory remission within six months/one year. Recent data estimate the remission rate of 65-80% for type II AIH (12), and higher than 80% for small ducts primary cholangitis (13-15). On the other hand, the frequency of relapses is still high in children so many trials with different immunosuppressive strategies have been performed (cyclosporine, tacrolimus, mycophenolate mofetil) (1).

At the present AIH–PSC diagnosis is still difficult because of the lack of clear criteria, rarity, and differences between children and adults (2, 5, 11). As reported in the most recent works, it remains unclear whether the overlap syndromes form distinct entities or they are only variants of one major autoimmune liver disease (7, 11). 

Standardization of overlap syndromes’ diagnosis is still lacking, especially when they present with “non distinctive” features of PSC or PBC, as in our patient. In fact, in our case, the clinical and histological signs of PSC were not confirmed by MR-cholangiography, thus leading to diagnosis of AIH - small duct PSC overlap. Is important to underline that our patient presented a type II AIH-PSC overlap, in contrast to what has been mainly observed in other reports (8, 10), in which type I AIH is the form mostly observed in the overlap syndromes. Furthermore either PBC or PSC may show auto-antibodies (ANA, SMA) or severe lymphocytic interface hepatitis even though they are not AIH (7, 11). 

The clinical case we presented add new data on this controversial point, taking in consideration that more reports could be useful to better define the limits of these autoimmune liver diseases and to improve the management of these patients.

## References

[1] Strassburg CP (2012). Autoimmune hepatitis: new guidelines, new therapies.. Dig Dis..

[2] Czaja AJ (2013). The overlap syndromes of autoimmune hepatitis.. Dig Dis Sci..

[3] Alvarez F, Berg PA, Bianchi FB, Bianchi L, Burroughs AK, Cancado EL (1999). International Autoimmune Hepatitis Group Report: review of criteria for diagnosis of autoimmune hepatitis.. J Hepatol..

[4] Hennes EM, Zeniya M, Czaja AJ, Pares A, Dalekos GN, Krawitt EL (2008). Simplified criteria for the diagnosis of autoimmune hepatitis.. Hepatology..

[5] Mieli-Vergani G, Vergani D (2011). Autoimmune liver diseases in children - what is different from adulthood?. Best Pract Res Clin Gastroenterol..

[6] Bellomo-Brandao MA, Costa-Pinto EA, De Tommaso AM, Hessel G (2006). Clinical and biochemical features of autoimmune hepatitis in 36 pediatric patients.. Arq Gastroenterol..

[7] Boberg KM, Chapman RW, Hirschfield GM, Lohse AW, Manns MP, Schrumpf E (2011). Overlap syndromes: the International Autoimmune Hepatitis Group (IAIHG) position statement on a controversial issue.. J Hepatol..

[8] Gregorio GV, Portmann B, Karani J, Harrison P, Donaldson PT, Vergani D (2001). Autoimmune hepatitis/sclerosing cholangitis overlap syndrome in childhood: a 16-year prospective study.. Hepatology..

[9] Mieli-Vergani G, Heller S, Jara P, Vergani D, Chang MH, Fujisawa T (2009). Autoimmune hepatitis.. J Pediatr Gastroenterol Nutr..

[10] Olsson R, Glaumann H, Almer S, Broome U, Lebrun B, Bergquist A (2009). High prevalence of small duct primary sclerosing cholangitis among patients with overlapping autoimmune hepatitis and primary sclerosing cholangitis.. Eur J Intern Med..

[11] Durazzo M, Premoli A, Paschetta E, Belci P, Spandre M, Bo S (2013). Overlap syndromes of autoimmune hepatitis: an open question.. Dig Dis Sci..

[12] Czaja AJ (2002). Treatment of autoimmune hepatitis.. Semin Liver Dis..

[13] Broome U, Glaumann H, Lindstom E, Loof L, Almer S, Prytz H (2002). Natural history and outcome in 32 Swedish patients with small duct primary sclerosing cholangitis (PSC).. J Hepatol..

[14] Angulo P, Maor-Kendler Y, Lindor KD (2002). Small-duct primary sclerosing cholangitis: a long-term follow-up study.. Hepatology..

[15] Bjornsson E, Boberg KM, Cullen S, Fleming K, Clausen OP, Fausa O (2002). Patients with small duct primary sclerosing cholangitis have a favourable long term prognosis.. Gut..

